# Leadership during airway management in the intensive care unit: A video-reflexive ethnography study

**DOI:** 10.3389/fmed.2023.1043041

**Published:** 2023-02-16

**Authors:** David J. Brewster, Warwick W. Butt, Lisi J. Gordon, Mahbub A. Sarkar, Jonathan L. Begley, Charlotte E. Rees

**Affiliations:** ^1^Intensive Care Unit, Cabrini Hospital, Melbourne, VIC, Australia; ^2^Monash Centre for Scholarship in Health Education, Faculty of Medicine, Nursing and Health Sciences, Monash University, Clayton, VIC, Australia; ^3^Central Clinical School, Faculty of Medicine, Nursing and Health Sciences, Monash University, Clayton, VIC, Australia; ^4^Royal Children’s Hospital, Melbourne, VIC, Australia; ^5^Centre for Medical Education, School of Medicine, University of Dundee, Dundee, Scotland, United Kingdom; ^6^School of Health Sciences, College of Health, Medicine and Wellbeing, University of Newcastle, Callaghan, NSW, Australia

**Keywords:** leadership, intensive care unit, airway, video reflective ethnography, intubation, simulation

## Abstract

Effective leadership is crucial to team performance within the intensive care unit. This novel study aimed to explore how staff members from an intensive care unit conceptualize leadership and what facilitators and barriers to leadership exist within a simulated workplace. It also aimed to identify factors that intersect with their perceptions of leadership. This study was underpinned by interpretivism, and video-reflexive ethnography was chosen as the methodology for the study. The use of both video recording (to capture the complex interactions occurring in the ICU) and team reflexivity allowed repeated analysis of those interactions by the research team. Purposive sampling was used to recruit participants from an ICU in a large tertiary and private hospital in Australia. Simulation groups were designed to replicate the typical clinical teams involved in airway management within the intensive care unit. Twenty staff participated in the four simulation activities (five staff per simulation group). Each group simulated the intubations of three patients with hypoxia and respiratory distress due to severe COVID-19. All 20 participants who completed the study simulations were invited to attend video-reflexivity sessions with their respective group. Twelve of the 20 participants (60%) from the simulations took part in the reflexive sessions. Video-reflexivity sessions (142 min) were transcribed verbatim. Transcripts were then imported into NVivo software for analysis. The five stages of framework analysis were used to conduct thematic analysis of the video-reflexivity focus group sessions, including the development of a coding framework. All transcripts were coded in NVivo. NVivo queries were conducted to explore patterns in the coding. The following key themes regarding participants’ conceptualizations of leadership within the intensive care were identified: (1) leadership is both a group/shared process and individualistic/hierarchical; (2) leadership is communication; and (3) gender is a key leadership dimension. Key facilitators identified were: (1) role allocation; (2) trust, respect and staff familiarity; and (3) the use of checklists. Key barriers identified were: (1) noise and (2) personal protective equipment. The impact of socio-materiality on leadership within the intensive care unit is also identified.

## 1. Introduction

Effective leadership in healthcare is important as it is known to optimize team performance (1–3). This is especially crucial in the complex environment of the intensive care unit (ICU) (3, 4). Existing evidence that underpins our understanding of leadership in the ICU is commonly discussed in an individualist fashion, similar to how it is conceptualized in the broader healthcare literature (4). Leadership within the healthcare environment has been conceptualized in different ways, with four discourses of leadership being described: individualist; relational; contextual; and complexity (1). In this way, leadership may be defined by the actions and styles of individuals (individual discourse), the leader-follower relationship (relational discourse), or how a context determines the behavior of leaders (contextual discourse). Finally, an emergent process of leadership can be described within an adaptive system (complexity discourse) (1).

Healthcare leadership has also been described by different dimensions, defined as leadership conceptualizations (1). A recent integrative review of the literature exploring leadership in the ICU identified two dominant discourses (individual and relational) and nine central dimensions (4). Dimensions such as role allocation, clinical skills, and communication skills defined leadership within the ICU as well as leader behaviors such as decision-making, being calm in a crisis, or being approachable, and traditional hierarchies (4). This integrative review highlighted a significant lack of literature relative to leadership and followership within the ICU, recommending that future research fill the gap by exploring ICU members’ experiences of leadership, as well as the key facilitators and barriers of leadership within this environment (4). This research could allow a richer understanding of how leadership is enacted in the context of an ICU. It may also facilitate further research into improving specific patient or staff-related outcomes attributed to leadership within this environment.

The ICU environment is unique, and the leadership dimensions may differ from other environments. Critically unwell patients are cared for by multi-disciplinary teams in an environment which can be very busy and often chaotic due to inadequate staffing or a life-threatening emergency or an elective procedure with adequate staff and time for preparation and planning. The COVID-19 pandemic has also had a significant impact on this environment, through the increased utilization of personal protective equipment (PPE), changes to the physical ICU environment, as well as the need for additional staff training and new team dynamics (5). Leadership within this complex environment is known to also face the challenges of certain historical influences, particularly those of hierarchy and gender (4, 6). Disciplinary hierarchies describe the traditional power imbalance between doctors and nurses or between senior and junior staff within the medical profession, which can make any collaborative approach to leadership more challenging (7). The role of gender in medical leadership has also been broadly discussed in the literature. This has not only been limited to emergency and crisis leadership, but also in formal leadership positions within the ICU (6). Leadership styles of men and women may be different, with male leadership associated with a more traditional authoritative style and female leadership being associated with more inclusiveness (8). The latter may be less likely to be recognized by medical teams as ‘leadership’ (8).

Currently, little is known about how ICU staff conceptualize leadership within the ICU and what dimensions, such as gender and hierarchy, impact leadership and followership in this context. Furthermore, whether particular barriers or enablers to leadership exist within this complex environment is also unknown. To our knowledge, no studies report the impact of the ICU environment on leadership and followership among ICU teams. In particular, how socio-materiality impacts leadership (see Table 1 for a glossary of key qualitative and theoretical terms), whereby socio-materiality describes how human beings, physical objects, and physical environments interact (13).

**Table 1:** Glossary of qualitative and theoretical terms.

Term	Meaning
Video-reflexive ethnography *(VRE)*	A research methodology that is both “ethnographic, in that video captures participants in their ‘natural’ working environment, and is ‘reflexive’, in that it involves participants exploring as a group what was captured on the video footage” (9).
Socio-materiality	A “focus on materials as dynamic and enmeshed with human activity in everyday practices” (10).
Interpretivism	An understanding through research that “looks for culturally derived and historically situated interpretations of the social life-world” (11).
Abductive coding	A coding process in qualitative research by which researchers “start with a deductive codebook and through the process of coding, build the codebook and, by extension, build theory by developing data-driven inductive codes” (12).

Ethnography is a qualitative research method which involves the observational study of people in their own environment. Video-reflexive ethnography *(VRE)* refers to the practice of filming professionals at work and using the footage to allow scrutiny and discussion about their work and behaviors at reflexive sessions (14). This interpretive tool has been shown to improve staff understanding of their behaviors, as well as to allow further improvement in their practice to enhance patient safety (14–16). VRE can therefore be used as an interpretive method to understand the environment and how staff behave. VRE has been used previously in this way to study staff communication in the clinical ICU setting, and as an interpretive research tool exploring leadership within broader healthcare (9, 17). It has yet to be utilized to look at leadership within ICU teams. The use of VRE by ICU teams as a research tool requires video of their practice within the ICU environment. However, in the face of the COVID-19 pandemic, video of real-life situations in the ICU has been challenging.

Simulation can be used as a surrogate to facilitate VRE to better understand staff performance in the ICU. Simulation-based staff training in healthcare has shown improvements in procedural performance, teamwork, and communication (18). Within the ICU, simulation-based team training has been demonstrated to facilitate clinical learning and positively alter staff behaviors (19). During the recent COVID-19 pandemic, the use of simulation-based team-training for airway management in ICU was ubiquitous, with one study demonstrating its use in 97% of Australian and New Zealand ICUs (20). Simulation training often occurs within a designated simulation training center, which aims to replicate the environment of a clinical space. However, *in-situ simulation* refers to simulation training done within the actual clinical space, potentially providing improved fidelity, cost-effectiveness, and staff familiarity with devices and their environment (21, 22).

This study was designed to video the simulations of airway management within a busy ICU and use VRE to further investigate leadership within the ICU.

It aims to address the following research questions (RQs):

1. How do ICU staff members conceptualize leadership through their reflections on the simulated ICU?
2. What are the ICU staff members’ perceptions of facilitators/barriers to leadership within the simulated ICU?
3. What factors intersect with the ICU staff members’ perceptions of leadership in the simulated ICU?

## 2. Materials and methods

### 2.1. Study design

This study was underpinned by interpretivism, understanding that multiple perspectives of reality exist which the research will investigate. VRE was chosen from an interpretive perspective as the methodology for the study to answer the RQs (23). Given that leadership is complex and enacted through dynamic interactions underpinned by communication (24), our research is grounded in social constructionism, where knowledge and experiences of leadership are created through relationships and shared social experiences (25). Therefore, we accept that complex and multiple truths exist, and we aimed to use VRE to understand them to address our RQs.

The use of both video recording (to capture the complex interactions occurring in the ICU) and team reflexivity (which provides further social interactions among participants) provides opportunities for repeated analysis of those interactions by the research team. VRE makes the complex environment of the ICU and the relationships for which leadership depend upon visible to the research team and the participants. It also allows for detailed and repeated analysis by the participants to drive understanding of their environment and work practices (14, 26).

Ethics was obtained from the hospital Human Research Ethics Committee (06–04–03-21).

### 2.2. Sampling and recruitment

Purposive sampling was used to recruit participants from an ICU in a large tertiary and private hospital in Melbourne, Australia. Simulation groups were designed to replicate the typical clinical teams involved in airway management within the ICU. Twenty-two ICU staff were invited *via* email to participate in this study. Twenty staff consented (91%) and participated in the four simulation groups (five staff per simulation group). Written consent was obtained by the lead author for: 1. Video recording during simulation; 2. Use of video and photos from the simulation for publication purposes; and 3. Participation in the video-reflexivity sessions. All invited participants were provided with specific participant and relevant ethics information and signed consent forms for participation. All 20 participants who completed the study simulations were invited to attend video-reflexivity sessions with their respective group. Twelve of the 20 participants (60%) from the simulations took part in the reflexive sessions.

Nurses were defined as “senior” when having greater than 7 years of ICU nursing experience, whereas junior nursing staff were categorized as those with less than 7 years of ICU nursing experience. All consultant ICU medical staff had completed fellowship training with the College of Intensive Care Medicine (CICM) of Australia and New Zealand. Trainee medical staff had yet to complete fellowship training. The composition of three groups (in relation to seniority of staff) reflected “in-hours practice” (groups 1, 3, and 4). One team reflected “after-hours” practice (group 2). Descriptions of the individual participants are in Table 2.

**Table 2:** Participant information.

Participant	Profession	Seniority	Gender
Group 1			
N1	Nurse	Senior	Female
N2	Nurse	Senior	Female
N3	Nurse	Junior	Female
D1	Doctor	Consultant	Male
D2	Doctor	Trainee	Male
Group 2			
N4	Nurse	Senior	Female
N5	Nurse	Senior	Female
N6	Nurse	Senior	Female
D3	Doctor	Trainee	Female
D4	Doctor	Trainee	Female
Group 3			
N7	Nurse	Senior	Female
N8	Nurse	Senior	Female
N9	Nurse	Junior	Female
D5	Doctor	Consultant	Male
D6	Doctor	Trainee	Female
Group 4			
N10	Nurse	Senior	Female
N11	Nurse	Senior	Female
N12	Nurse	Junior	Male
D7	Doctor	Consultant	Female
D8	Doctor	Trainee	Male

Four groups of ICU staff were assembled to complete a total of 12 *in-situ* simulations, where each group completed simulations of three different contexts/phases of airway management within the ICU.

### 2.3. Data collection

**Figure 1:**
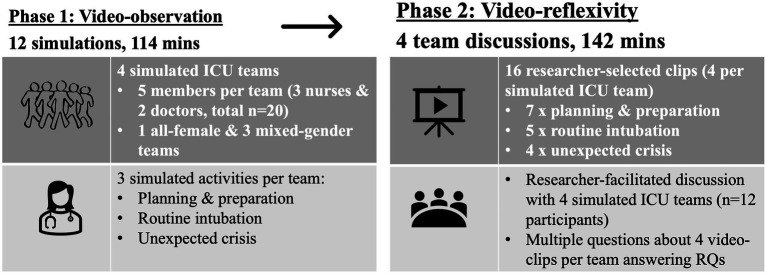
Overview of data collection.

The first author, who is a practicing intensive care specialist from within the workplace where the study was undertaken, collected all data. Data were collected in two phases: video observation phase and video-reflexivity phase (see Figure 1 for an overview of data collection phases).

#### 2.3.1. Video-observation phase

Each group simulated the intubations of 3 patients with hypoxia and respiratory distress due to severe COVID-19 (see Supplementary material 1). All participants wore airborne PPE including goggles/glasses, masks, gloves, gowns, and face shields, consistent with normal clinical practice during all simulations. The simulations occurred inside a busy working ICU both outside and inside a negative pressured room. Each group completed simulations involving three phases of airway management representing different clinical activities and contexts:

1. Planning and preparing for airway management (occurring outside the simulated patient’s room).
2. Performance of a routine intubation procedure (occurring inside the simulated patient’s room) *immediately* following phase 1.
3. Management of an unexpected crisis (occurring inside the room) *filmed a few minutes after* the start of 1 and 2.

The unexpected crises simulated included one of the following:

1. A power failure inside the room (groups 2 and 3)
2. Conscious collapse of a medical practitioner responsible for intubation during the procedure (prior to its safe completion) (group 4)
3. Failure of a safe completion of the intubation procedure (group 1)

**Figure 2:**
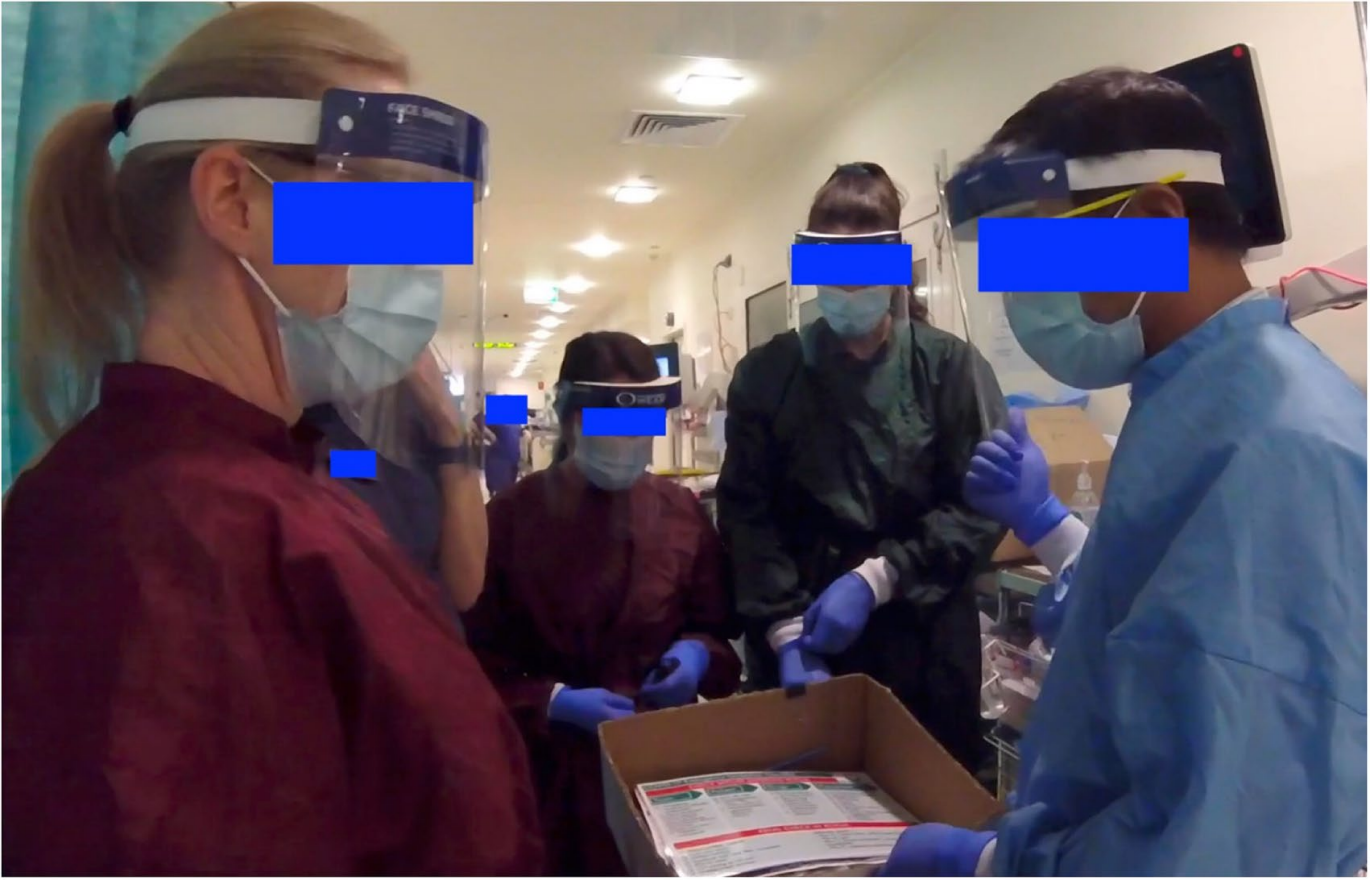
Photo of simulated planning by group 3 (From left to right N7, N8, N9, D6, D5).

**Figure 3:**
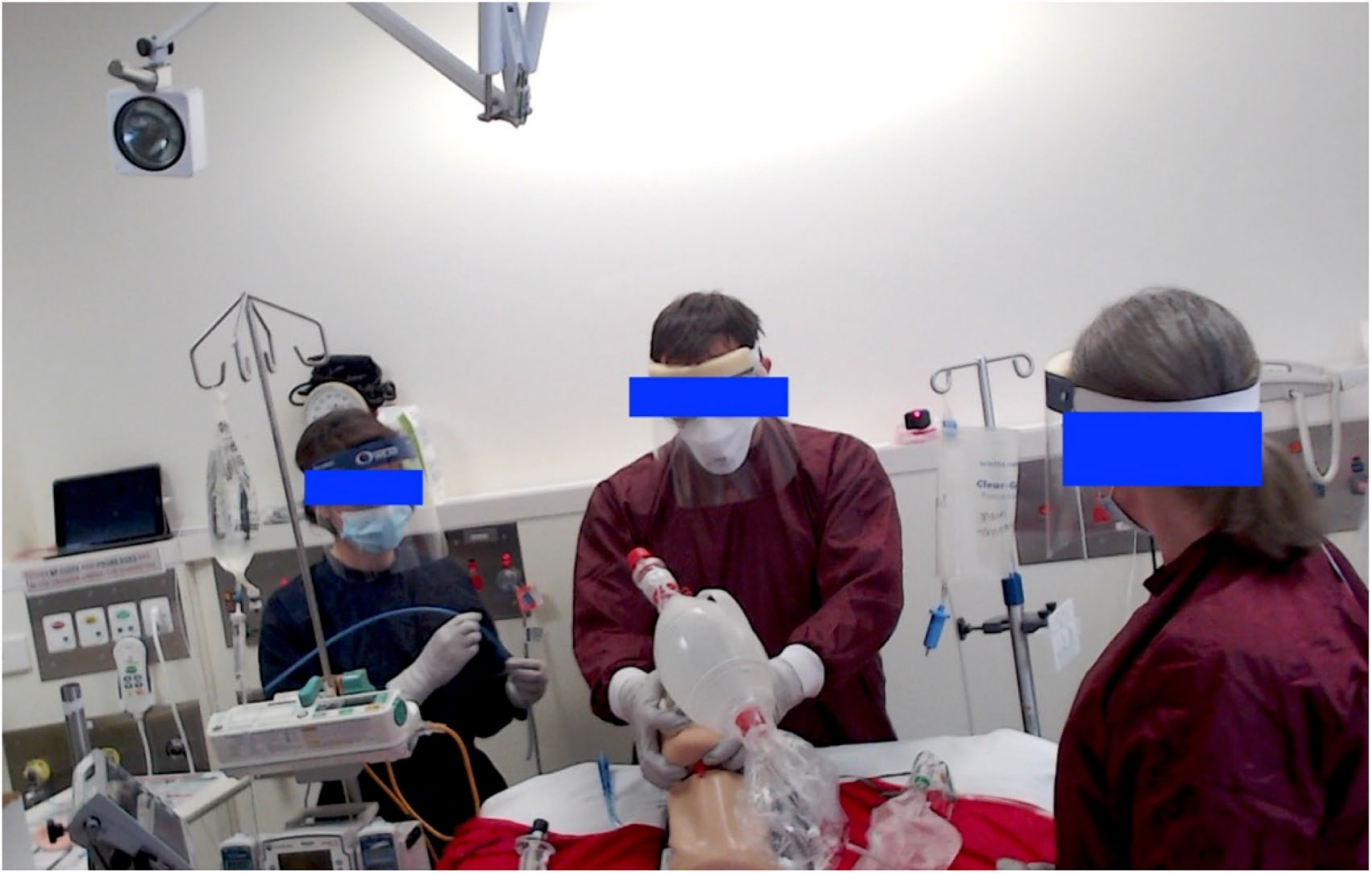
Photo of simulated procedure by group 4 (From left to right: N11, D8, D7).

**Figure 4:**
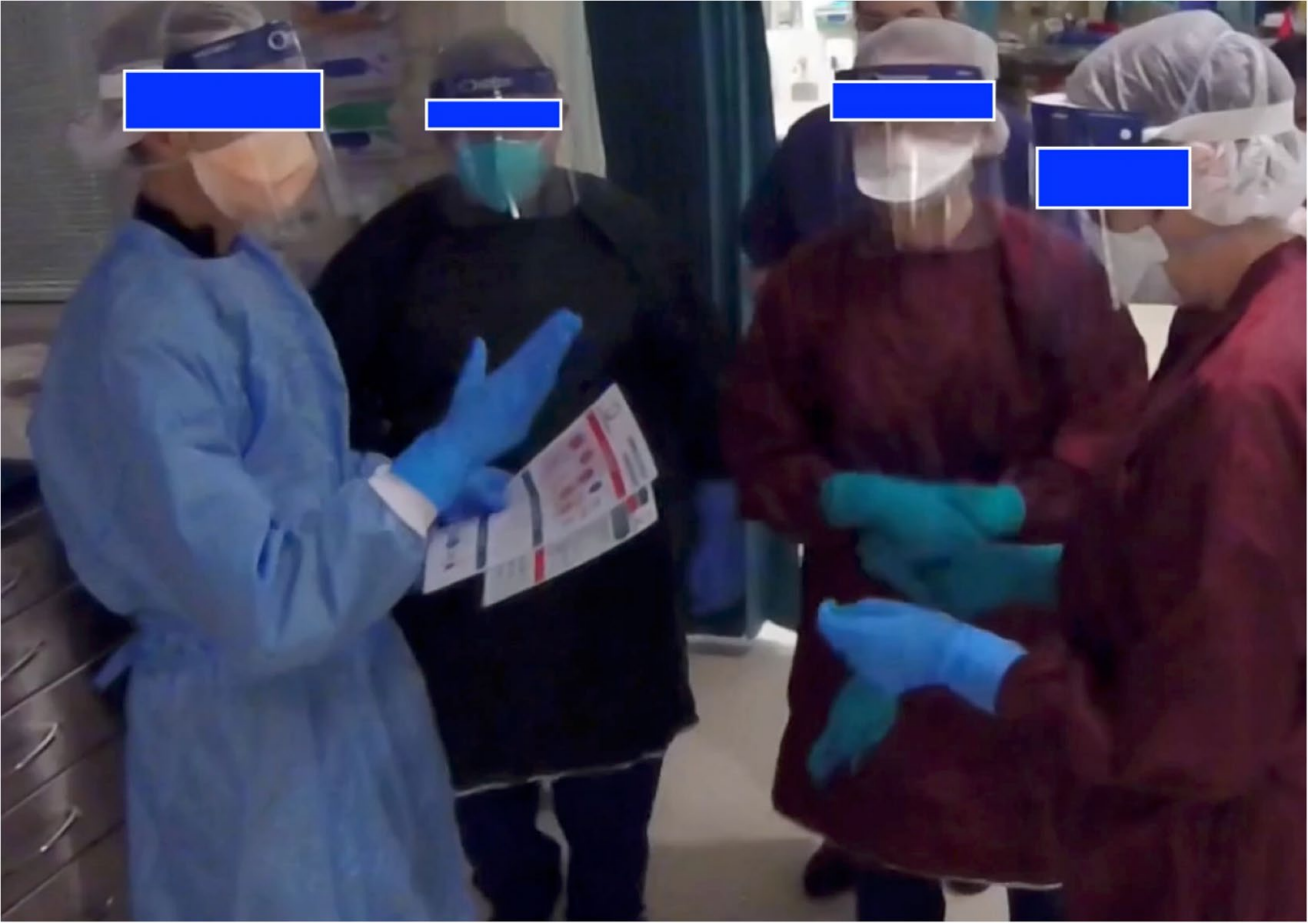
Role allocation occurring during clip 2 (referred to in quote 19).

All simulations were recorded on two separate video cameras. One was a fixed camera in the corner of the simulated patient’s room. The other was a roving GoPro camera controlled by the first author. In total, 114 min of simulated activities was recorded. The first author chose 16 video clips (see Table 3) to use for the four VRE sessions, sharing four clips with each group (see Table 3 for details). Clips were chosen by the first author and confirmed after review, by a second author (WB) who is also a senior intensive care clinician-researcher. Clips were chosen in this way to take an interpretive approach to the research study; to maximally understand the data relevant to the RQs. Clips were also chosen to represent diversity across the three activity phases of the scenarios (including different clinical contexts) and were identified as good trigger materials for discussions about leadership, thereby helping to answer the study RQs (see Figures 2–4).

**Table 3:** A summary of video clips used for reflexivity.

Group	Clip	Clinical phase/context	Duration (seconds)	Summary
1	1	Planning	48	Team meet outside room and agree to plan an intubation. Roles are allocated by senior doctor (D1) and senior nurse (N1). Staff attempt to put on PPE.
	2	Planning	56	Team huddle in PPE to read a checklist before entering the simulated patient’s room.
	3	Procedure	40	Intubation is performed by junior doctor (D2) with assistance of senior nurse (N2) and direction of senior doctor (D1) (allocated role of team leader). Senior doctor (D1) helps complete the intubation task.
	4	Crisis management (Crisis scenario 3)^*^	66	Unexpected failure of intubation by junior doctor (D2). Team advised by senior doctor (D1) for junior doctor to abandon attempts at intubation and change to “plan B” and insert laryngeal mask airway device to rescue ventilate the simulated patient.
2	5	Planning	45	Team meets outside room and agree to plan an intubation. Roles are allocated by junior doctor (D3) and senior nurse (N4). Checklist is read by group.
	6	Planning	35	Role allocation and planning continues to occur in a group forming a circle around a checklist. Second junior doctor (D4) speaks up to acknowledge a lack of confidence with the scenario given her perceived lack of experience.
	7	Procedure	52	Difficult procedure where first attempt at intubation is unsuccessful by the junior doctor. Same junior doctor (D3) decides to insert laryngeal mask airway (LMA) device to allow time to oxygenate the patient and think about her next steps.
	8	Crisis management (Crisis scenario 1)^*^	62	Unexpected power failure within the ICU (lights go out) while team member attempts intubation. Junior doctor (D3) leads team in assembling battery powered lighting for the room and completing the task of intubation and rescue the crisis.
3	9	Planning	66	Team meets outside room to plan an intubation. Roles are allocated by senior doctor (D5) only and clarified by the others. Staff are standing in a circle and all wearing PPE.
	10	Planning	42	Team discuss plan for intubation one more time inside the simulated patient’s room while setting up equipment. Senior doctor (D5) answers questions from junior doctor (D6) about tasks required for the procedure.
	11	Procedure	44	Routine intubation procedure commences. Tasks shared between senior doctor (D5) and senior nurse (N7).
	12	Crisis management (Crisis scenario 1)*	60	Unexpected power failure within the ICU (lights go out) while team attempting intubation. Senior doctor (D5) and senior nurse (N7) instruct team to assemble battery powered lighting for the room and complete the intubation task.
4	13	Planning	60	Team huddle in a circle (all in PPE) and read through checklist and discuss procedure. Junior doctor (D8) begins leading the process but senior doctor (D7) takes over leading the process during the verbalizing of the plan for the procedure by the junior doctor (D8).
	14	Procedure	53	Team starts the intubation attempt. Lots of dialogue between junior and senior doctors (D8 and D7). Junior and senior nursing staff are busy preparing equipment (N11 and N12). Team members have to re-position multiple pieces of equipment either obstructing the action or out of position (including the bed, video laryngoscope monitors and oxygen apparatus) as they attempt to start oxygenation and make the environment safer to work in.
	15	Procedure	43	Team pause after the start of the procedure to clarify one final time the next steps in the procedure. Senior doctor (D7) clarifies everyone is ready to continue to the next step. Junior doctor (D8) verbalizes the plan while simultaneously also oxygenating the patient with a self-inflating resuscitation bag and mask.
	16	Crisis management (Crisis scenario 2)^*^	39	Junior doctor (D8) responsible for intubation collapses with chest pain just after patient receives paralyzing drugs but before intubation can be attempted. Senior nurse (N11) abandons task to attend to junior doctor on floor. Senior doctor (D7) takes over the procedure and re-allocates roles to other staff to safely complete the procedure.

Crisis scenario 1 = power failure; Crisis scenario 2 = Conscious collapse of a medical practitioner; and Crisis scenario 3 = failure of intubation.

#### 2.3.2. Video-reflexivity phase

The 12 participants at the four discrete reflexive sessions were asked to watch the selected video clips from their simulations and discuss them as a group with respect to the overarching RQs. Due to ongoing COVID-19 restrictions, the video-reflexivity sessions were conducted on Zoom *(Zoom Video Communications Inc., California, USA, 2016)*. The sessions were facilitated by the first author. These reflexivity sessions were audio-recorded. During these semi-structured sessions, participants were asked various questions including:

1. What do you see in this clip?
2. How is leadership / followership enacted and why is it this way? What makes you think that?
3. How do you feel leadership/followership in this clip relates to leadership in the ICU in general?
4. What (if any) were the barriers to leadership in this clip?
5. What (if any) were the facilitators to leadership in this clip?
6. Does this clip relate to any of your past experiences of leadership in the ICU? Please describe.
7. Is there anything else you want to say about leadership in this clip?

Free conversation between participants relevant to the research questions was encouraged. Reflexivity sessions ranged in duration from 30 to 42 min, with an average of 35.5 min across the four groups. This provided a total of 142 min of video-reflexivity sessions that were transcribed verbatim. Transcription was initially conducted by Otter.ai software *(Otter.ai, Los Altos California, 2022)*, then checked (with errors corrected) by the lead author. Transcripts were then imported into NVivo software version 12 *(QSR International Pty Ltd, Version 12, 2018)* for analysis.

### 2.4. Data analysis

The study employed a thematic analysis approach drawing on a previously developed coding framework, while maintaining openness to new themes based on the data.

The five stages of framework analysis were used to conduct thematic analysis of the video-reflexivity focus group sessions (27).

*Familiarization*. All transcripts were read by the first author (DB). One of the transcripts was read by each of four other authors (WB, LG, MS, CR).

*Development of coding framework*. This study used an abductive approach to develop a coding framework (28). Using a previous inductively developed coding framework as a starting point (4), further development of that coding framework was done (on the basis of the data and our study research questions). The first author (DB) identified additional themes in all transcripts to add to the previously published coding framework specific to leadership within the ICU (4). These themes were recorded and then sorted into codes and added to the coding framework. Four other authors (WB, LG, MS, and CR) read one transcript each to also identify additional themes to allow quality assurance of the new coding processes. This framework (see Supplementary material 2, with new codes highlighted) was imported into the NVivo software to help facilitate the analysis of the VRE transcripts.

*Indexing*. All transcripts were coded in NVivo by the lead author and a selection of coding was checked by the second author (JB).

*Charting*. NVivo queries were conducted to explore patterns in the coding. NVivo provides counts of the number of quotations coded to each theme/sub-theme, so it was possible to first identify the dominance of certain themes/sub-themes across the whole dataset, plus identify the dominance of themes based on specific participants’ contributions (junior/trainee versus senior staff, medical or nursing staff) or based on reflection on certain phases of the simulated scenarios (e.g., planning and preparation, versus routine procedures versus unexpected crises). Hierarchical charts demonstrated the distribution of coding frequencies within the coded quotations from the VRE transcripts. This enabled the researchers to identify key themes relevant to our RQs. Analysis of tabulated coding frequencies was done in NVivo with heat mapping. This was used to further explore which themes were identified as more relevant to certain contexts and/or from the responses of specific participants.

The co-occurrence of themes was explored using matrix coding queries, which demonstrated the intersections between coded quotations for themes/sub-themes and different participants or clinical contexts.

*Interpretation*. Themes were summarized and presented in quotations, hierarchical charts, and tables. Within tables, color coding of frequencies or coded quotations (heat maps) were used. Heat maps show varying density of data when comparing themes/sub-themes by groups. These were discussed among the research team during interpretation. Key themes are presented in narrative form with illustrative quotes, allowing us to answer the RQs.

### 2.5. Team reflexivity

Team reflexivity acknowledged differences among our six researcher backgrounds, theoretical positioning and experiences. Our team included four males and two females, three intensive care specialists (all with research experience in airway management), one physiotherapist with 10  years of ICU experience. One of us has a background in psychology. Three of us are health professionals and education researchers with expertise in qualitative and/or leadership research. Two of us have previous experiences employing VRE as a methodology and one of us has experience video-recording *in-situ* simulations. This diversity allowed for rich debate on the meaning of the data that was captured and analyzed, as well diversity contributed to ensuring the rigor of the study.

## 3. Results

Codes were added to the coding framework (see abductive coding framework in Supplementary material). Most notably, some new codes referred to the role of socio-materiality in the ICU. The significance of these interactions was noted initially by the researchers in their viewing of the simulations, as a theoretical/philosophical construct, and subsequently in their analysis of the transcripts of the reflexivity sessions.

Individual quotations (*n* = 380) from video-reflexivity session transcripts were coded to various codes across the dataset. These quotations were identified from a relatively evenly distributed number of reflections by medical and nursing staff (47% vs. 53%) and in reference to the three scenario activity phases (phase 1 = 31%, phase 2 = 32% and phase 3 = 37%). Senior staff were responsible for most of the quotations (73%), comprising 10 of the 12 participants in the reflexive sessions.

The following key themes to leadership within the ICU were identified to answer the overarching RQs:

### 3.1. RQ1: How do ICU staff members conceptualize leadership through their reflections on the simulated ICU?

Leadership within the ICU was conceptualized by the participants in a variety of ways during the reflexive sessions. The most dominant themes are presented below and in Tables 4–7.

**Table 4:** Illustrative quotes (conceptualizations of leadership).

D5 (consultant doctor, group 3, clip 10)	*“But I think at the nitty gritty, it would be a shared role with people bouncing off each other.”* *(Quote 1)*
N5 (experienced nurse, group 2 final comment, no specific clip)	*“Somebody needs to be leading. It is a collaborative thing, but it’s not a leadership community. It’s one person.”* *(Quote 2)*
N5 (experienced nurse, group 2 final comment, no specific clip)	*“Obviously, a lot of it is clear communication. And it’s, and when I say communication, it’s knowing when to shut up as well.”* *(Quote 3)*
N2 (experienced nurse, group 1, clip 1)	*“He (D1) is always very clear about what he wants done. In a clear, calm, concise way.”* *(Quote 4)*
D3 (trainee doctor, group 2, clip 7)	*“My experience is that men are naturally perceived as the leader.”* *(Quote 5)*
D3 (trainee doctor, group 2, clip 7)	*“In other departments, a junior male will often be sought after over a senior female to lead a scenario. Gender plays heavily and I think … females must really work on performance in order to be perceived as the leader.”* *(Quote 6)*
N5 (experienced nurse, group 2, clip 7)	*“I think sometimes we feel a bit more comfortable with the women team leaders because we feel like they are more likely to include us…”* *(Quote 7)*

**Table 5:** Intensive care unit (ICU) staff members’ perceptions of key facilitators and barriers to leadership.

Key facilitators	Role allocation	Trust, respect and staff familiarity	Checklists
Key barriers	Noise	PPE

**Table 6:** Illustrative quotes (facilitators of leadership).

D3 (trainee doctor, group 2, clip 5)	*“You need to be very clear as the leader and the team gains confidence from clear role allocation. So, it’s your responsibility as the leader to ensure that that’s done properly. There’s no confusion.”* *(Quote 8)*
N5 (experienced nurse, group 2, clip 5)	*“When it’s done well, it’s one person allocating”* *(Quote 9)*
D1 (senior doctor, group 1, clip 1)	*“A pretty privileged position, having N1 and N2 (who are) very senior nursing staff that I have worked with a lot and trust and know their capabilities. That really helps as a leader.”* *(Quote 10)*
D5 (senior doctor, group 3, clip 9)	*“I would say the familiar environment and familiarity with the staff. Because we have known each other we know each other’s skills and strengths. So that makes it easier.”* *(Quote 11)*
N1 (senior nurse, group 1, clip 1)	*“I think respect has a lot to do with enabling multiple leaders.”* *(Quote 12)*
N10 (senior nurse, group 4, clip 15)	*“Checklists have been very important in making sure things aren’t getting missed and that everything is done as safely as possible for the patient.”* *(Quote 13)*
D5 (senior doctor, group 3, clip 11)	*“So you just go through the checklist, make sure I’ve got everything here, and everyone knows what they are doing.”* *(Quote 14)*
N10 (senior nurse, group 4, clip 13)	*“And there was the checklist that was read slowly and deliberately, which allowed time for team members to speak up if needed to clarify any issues.”* *(Quote 15)*

**Table 7:** Illustrative quotes (barriers to leadership).

N10 (senior nurse, group 4, clip 14)	*“Noise is always a potential barrier to leadership. Obviously, just with all of the beeps of the machines, if you add in people talking elsewhere, as well, it can become quite difficult for leadership to be maintained, and for control of the situation to stay with the leader.”* *(Quote 16)*
D5 (senior doctor, group 3, clip 10)	*“And the same issue with the noise, the phone ringing, lots of distractions in terms of leadership.”* *(Quote 17)*
N2 (senior nurse, group 1, clip 2)	*“I was a bit distracted by trying to get my gloves on.”* *(Quote 18)*
N1 (senior nurse, group 1, clip 2)	*“I think one of the big major barriers, obviously, in that situation is the PPE. [commenting on planning phase and role allocation – see Figure 4] It makes it very, it makes it harder to hear, you do not see their (the leader’s) facial expressions… you cannot lip read if there’s background noise.”* *(Quote 19)*

#### 3.1.1. Leadership is both a group/shared process and individualistic/hierarchical

The two dominant themes identified were leadership as a *group process* and as *hierarchy*. We found these leadership dimensions to be context-dependent. Differences in attitudes to leadership were reported from the planning phase before performing intubation, which was often referred to as a group process (quote 1), to the unexpected crisis management, more frequently described as individualistic (quote 2). During discussion of the crisis management, a more hierarchical and individualistic leadership model (of the most senior doctor taking charge) was described (predominantly by nursing participants). Hierarchy occurs most commonly in reference to a doctor leading a team of nursing staff and represents the most common individualistic leadership model described.

The participants described group leadership as a shared and distributive model, whereby multiple team members make decisions (including role allocation and clinical decisions) at the same time to prevent cognitive overload on one individual. This is most commonly described as occurring when a senior nurse and a doctor share the leadership.

#### 3.1.2. Leadership is communication

All groups reflected on communication as a key dimension of leadership. Many participants reflected on the need for communication to be clear and concise (quotes 3 and 4), as well as the need for silence from followers (quote 3). As a dimension, it was the third most abundant theme we identified.

#### 3.1.3. Gender is a key dimension within the ICU

One group (the all-female group) reflected on how their gender impacted past experiences of leadership. Note that gender as a key leadership dimension was not discussed in the other three (mixed-gender) groups. This all-female group discussed their past experiences in the ICU of male staff being perceived as the leader (quotes 5 and 6) in preference to female staff, with female leaders reportedly being more inclusive of other female team members in decision-making processes (quote 7). Further to this, this group acknowledged the struggles of females to be allocated and/or assume leadership roles within the ICU (quote 6).

### 3.2. RQ2: What are the ICU staff members’ perceptions of facilitators/barriers to leadership within the simulated ICU?

#### 3.2.1. Facilitators of leadership

##### 3.2.1.1. Role allocation

Quote 8 highlights understanding role allocation as both a key leadership behavior and a key facilitator to team performance. The act of allocating roles was seen by many participants as a leadership behavior and usually done by one person (quote 9). Followers also felt more confident in their performance when allocated a clear role.

##### 3.2.1.2. Trust, respect, and staff familiarity

All groups identified team trust and familiarity as key facilitators of leadership in the ICU. Knowing each other, and respecting others’ skills and knowledge was seen to create the platform for leadership (quotes 10 and 11). Respect was also seen as a key facilitator to distributed leadership occurring (quotes 12).

##### 3.2.1.3. Checklists

Participants indicated that the use of checklists to guide clinical decision-making was vital as a facilitator of leadership during airway management within the simulated ICU. Checklists were seen by the staff as a tool for ensuring patient safety (quote 13) and helping to familiarize staff with the correct processes and equipment to use (quote 14). They were also thought to be a tool allowing the team to stop, talk through a procedure, and allow followers to speak up if they had concerns or needed clarification of the process (quote 15).

#### 3.2.2. Barriers to leadership

##### 3.2.2.1. Noise

Participants identified noise as the most significant barrier to leadership, referring to noise from other staff (quote 16), as well as beeping machines and monitors (quotes 16 and 17). Participants in all groups commented on the competing chatter of sub-groups within the simulation scenarios, the ambient noise of ICU and the noise from equipment (such as alarms and the continuous beeping noise of the simulated patient monitor’s pulse oxygen saturation).

##### 3.2.2.2. PPE

PPE was commented on by staff to be a profound barrier to communication and leadership within the ICU. Participants described the distraction of having to put on their PPE during role allocation and planning (quote 18), as well as the clothing being a significant barrier to both verbal and non-verbal communication (quote 19).

### 3.3. RQ3: What factors intersect with the ICU staff members’ perceptions of leadership in the simulated ICU?

Matrix coding allowed for identification of how the above findings intersected with either the staff involved or the situational context. We found that:

Perceptions of leadership, being a *shared or individual process*, were context driven. The shared model was most discussed after watching video clips of simulated planning for a procedure, whereas individual and hierarchical leadership was described in reference to video clips of airway management during a crisis.

*Role allocation* was the most identified facilitator to leadership mentioned across all three phases of the scenarios and by all staff (both medical and nursing participants, as well as senior and junior staff). Role allocation is seen to be the key leadership act in all scenarios that allowed followers to feel confident in their performance.

Calm and *clear communication* was seen as most important during the performance of a procedure or management of crisis.

In terms of barriers to leadership, *noise from other staff* as a barrier to leadership was most identified primarily by senior nursing staff. However, all staff (senior, junior, medical, and nursing) referred to the noise of machines as a major barrier to leadership.

*PPE* was most frequently reported to be a barrier to leadership during reflection on the planning phases of airway management. It seemingly distracted staff during the role allocation process and inhibited them from understanding (both verbal and non-verbal) communication.

## 4. Discussion

This study used both *in-situ* simulation and VRE to better understand ICU staff members’ conceptualizations of leadership within the complex environment of their ICU. Through an interpretive lens, we found that participants largely spoke about leadership as group (shared) and hierarchy (individual) processes when triggered by different phases/contexts of the scenario (e.g., during the team planning or time critical performance of airway management respectively). The results of this study, therefore, demonstrate that leadership in the ICU is not viewed in a *one-size-fits-all* way by ICU staff. Indeed, context varies understandings of leadership. Group or shared leadership was described across all contexts of the simulations but was perhaps more likely to occur during the planning phase and the traditional individualistic hierarchal leadership model became more noticeable in crisis management. Calm and clear communication was also seen as an important leadership dimension within the ICU, especially during the performance of a procedure or in the context of managing a crisis. Female staff also reported that gender was a key underlying dimension to leadership within the ICU, with female doctors finding it more difficult to be perceived as leaders in the presence of male counterparts.

The dichotomy of leadership we have described within the ICU, being both a shared and individual process, is well described in other healthcare literature (1). However, our findings are new and important in the context of the ICU. Gender is known to be a significant issue within the ICU medical workforce (6). Albeit from one group of all-female staff, we also found that gender has been a key theme in some previous negative experiences relevant to leadership among female staff in the ICU, both medical and nursing. In particular, our female staff in this group reported leadership to be more difficult to be granted or accepted in the presence of male medical staff. Furthermore, as suggested in the previous literature based on research within the operating room, this study highlights that some female staff within the ICU have also experienced more inclusive leadership from female leaders (8).

Whichever leadership dimension is at the forefront of ICU clinicians’ minds, participants felt that key facilitators to leadership occurred across individual, relational, and organizational levels. At the individual level, role allocation was the most widely discussed facilitator of leadership. Clear role allocation was seen as a positive leader behavior and gave followers confidence to complete their tasks. Trust, respect, and familiarity with other staff was also reported to be a key relational facilitator to leadership in the ICU setting. Finally, organizational endorsement of checklists was another important perceived leadership facilitator by the staff.

The ICU environment is perhaps the greatest barrier to leadership. Machines and equipment were reported to be key causes of distraction, through noise, as well as the physical environment crowded with so many people. After the COVID-19 pandemic, a new barrier to leadership (i.e., PPE) is significant, especially in its effects on both verbal and non-verbal communication. According to the reflections of our participants, noise was seen as a significant distractor. Noise from machines, the background noise of a busy ICU and noise related to the conversation of other staff are all seen as marked barriers to team performance within the ICU. Noise within the ICU has previously been linked to negative outcomes, in particular poor sleep (29, 30). However, to our understanding, it has not been reported as a barrier to leadership within the ICU. These findings, both the facilitators and barriers to leadership within the ICU, have not to our knowledge been previously described within the literature.

### 4.1. Methodological strengths and challenges

There are many strengths to the innovative methodology of this study. A rigorous approach to team-based analysis of VRE data was employed using NVivo software. As a working specialist from within the ICU where the study was undertaken, the primary author (and data collector) had insider knowledge of the study environment. This facilitated comfort within the study environment and understanding of the language used by participants (31). The primary author also had the trust of the research participants. This may be reflected in the excellent response rate to the study invitation (91%). The familiarity of the staff with each other may have allowed them to feel safe in sharing their reflections and led to a vigorous discussion, helping the researchers to answer the RQs. The location of the *in-situ* simulations, being within the actual workplace of the participants, may have optimized the fidelity of the simulations and created a more realistic example of a clinical encounter. Finally, leadership practices were examined in a safe (simulated) setting, so research did not interfere with actual patient care.

There are a few limitations to our study to discuss. First, with this interpretive study, the sample size of the participants, the amount of data and diversity of the sample should be acknowledged as a limitation; influencing adversely the transferability of the findings to other contexts. However, efforts were made to select a sample of participants with a balance of clinical experience and gender that reflects the typical ICU workforce in Australia. Furthermore, the participants invited were aimed to reflect the balance of the allocated roles within the usual clinical workplace. Second, simulation data, despite however many efforts are made to maintain high fidelity, are still not real clinical practice. However, this was unavoidable given the research restrictions placed upon us by the COVID-19 pandemic. Third, our inclusion of pre-existing teams of participants, with established relationships and hierarchies, might mean that our study findings are not transferable to other ICU contexts, whereby airway teams are assembled in an impromptu fashion without established relationships and hierarchies. Fourth, the VRE methodology used for this study was through an interpretative lens only. Viewing the actions of a group by the participants themselves stimulated discussion among that group. Analysis of their reflexive discussions was done by the researchers to better understand leadership within the ICU. Further observations of the simulated videos and discussion by non-participants (ethical approval and participant consent permitting) may have identified additional leadership themes. Finally, as the study was conducted at only one hospital, there will always be concerns for transferability (as mentioned above). Given this study was undertaken at a large, private, metropolitan, and tertiary ICU, transferability of results to smaller or rurally located ICUs may be challenging. However, they should be transferable to other large Australian tertiary hospitals, which make up the majority of ICUs. Also, our participants work in other ICUs (public and private) further adding to the transferability of the results.

### 4.2. Future research

VRE was used in this study employing an interpretive lens, and further interpretivist research is needed to explore these issues in different contexts (e.g., smaller, rurally located ICUs), and in different countries with different educational systems in critical care. Moreover, further research using VRE in this area may choose to come from a critical inquiry perspective (23). This would allow researchers to determine if the knowledge gained from VRE could lead to changes in outcomes, either through altered staff performance and behaviors or patient-centered outcomes.

The effects of the ICU environment, particularly socio-materiality impacts, on leadership within ICU were significant. Noise, machines, PPE, and checklists all significantly affect leadership practices. A critical inquiry study could look at how to minimize the effect of perceived barriers and enhance the effects of any enablers on leadership.

Different leadership approaches, particularly an individualistic hierarchical style and a distinctly different shared or group interprofessional approach, were reported by participants in this study. This dichotomy of leadership approaches was described in different clinical contexts, including the planning phase prior to undertaking an intubation, as well as during the chaos and stress of an unexpected airway management crisis. The outcomes of either approach within the ICU need further research. Finally, the role of gender and its effects on both leadership approaches and staff confidence in enacting leadership could be further explored within the ICU setting.

This study has highlighted that VRE can enable ICU staff to visualize their leadership practices within their workplace. Simulation was realistic and participants’ experiences were consistent with real life. Future research should adopt a more critical inquiry approach to see if video and reflection could also improve their leadership practices.

## Data availability statement

The original contributions presented in the study are included in the article/Supplementary material, further inquiries can be directed to the corresponding author.

## Ethics statement

The studies involving human participants were reviewed and approved by Cabrini Hospital Research Governance (06–04–03-21). The patients/participants provided their written informed consent to participate in this study. Written informed consent was obtained from the individual(s) for the publication of any potentially identifiable images or data included in this article.

## Author contributions

Data collection was done by DB and JB. DB conducted coding framework development and checked by CR, WB, MS, and LG. Coding of transcripts was done by DB and results were discussed by all authors. DB was the primary author for the manuscript preparation. All authors contributed to the study design. All authors contributed to the article and approved the submitted version.

## Conflict of interest

The authors declare that the research was conducted in the absence of any commercial or financial relationships that could be construed as a potential conflict of interest.

## Publisher’s note

All claims expressed in this article are solely those of the authors and do not necessarily represent those of their affiliated organizations, or those of the publisher, the editors and the reviewers. Any product that may be evaluated in this article, or claim that may be made by its manufacturer, is not guaranteed or endorsed by the publisher.
