# Supplementary material for: Leadership during airway management in the intensive care unit: A video-reflexive ethnography study
Source: Front Med (Lausanne). 2023 Feb 16;10:1043041. doi: 10.3389/fmed.2023.1043041 (PMC9980339; doi:10.3389/fmed.2023.1043041)
Supplement: Supplementary file 1 [file Data_Sheet_1.docx]

Supplementary Material

# Simulation information

Equipment:

- Manikins suitable for intubation, surgical airway
- Oxygen mask, source and tubing
- COVID-19 intubation kits, containing:
- Endotracheal tubes, range of sizes
- Resuscitation bag, masks, oropharyngeal airways
- Laryngoscopes
- Suction catheter
- Sucker and tubing
- Laryngeal mask airways, range of sizes
- Bougie
- Capnograph
- Front of neck airway kit

Simulate a routine intubation in airborne personal protective equipment (PPE) in the intensive care unit (ICU).

Team of five ICU members to assemble and perform simulated intubation.
- 2 x medical staff and 3 x nursing staff

Participants are given a short clinical stem:

“You are called to intubate Peter, a 42-year-old man (70kg) with respiratory distress, hypoxia and a febrile illness in the ICU. Previous intubations are not documented. His vital signs are:
SpO2 is 92% on 15L/min oxygen
Respiratory rate 45
BP 95/50
HR 125
He has two large IV canulae.
His airway examination is unremarkable.
He had a known COVID exposure 4 days ago.”

# Abductive coding framework

*Note that this framework has been developed **abductively** from a published coding framework (Brewster et al. 2020) (4).

| **Leadership dimensions themes** | |
| --- | --- |
| **Leadership as:** | **Definition** |
| 1.1 Behaviour | Leadership is described explicitly as one or more behaviours with authors using the words behaviours or synonyms e.g. conduct, actions, etc. (e.g. decision-making behaviour, responsible behaviour etc.) |
| 1.2 Role allocation | Leadership is described by allocated job position within the ICU (e.g. supervisor, trainee, manager etc.) |
| 1.3 Profession | Leadership is described by health profession group (e.g. medical, nursing etc.) |
| 1.4 Hierarchy | Leadership is described by medical hierarchy within ICU (e.g. senior medical specialist as the leader or the saviour) |
| 1.5 Group process | This dimension is focused on team working both uni-professional and inter-professional (Including for example shared position and responsibility) |
| 1.6 Personality | Leadership is described by authors explicitly as personality characteristics or traits. Examples of this are participants talking about dominant personalities or individuals being “natural” leaders. |
| 1.7 Principles and values | The focus on leadership here is about the principles and values underpinning it. For example, participants might talk about a leader being fair, approachable, coaching and supportive, and allowing followers to develop and learn. |
| 1.8 Responsibility | Leadership is described as responsibility by the authors. For example, the person who has ultimate clinical responsibility within a given situation was perceived to be the leader. |
| 1.9 Clinical skills | Leadership is described in relation to the clinical skills of the leader such as being an expert in clinical performance. |
| 1.10 Communication skills | Leadership is described in relation to the communication skills of the leader such as their expert ability to communicate. |
| 1.11 Emergent | Leadership is described by its dynamic nature within the ICU team, with team members taking on leadership outside of their allocated roles within the ICU team. (So opposite to code 1.2) |
| 1.12 Management | Leadership is described as management explicitly by authors, with leadership and management seemingly conflated and thus including management roles such as organisational responsibilities |
| 1.13 Knowledge | Leadership is described in relation to the expert knowledge possessed by leaders |
| 1.14 Gender | Leadership is described in gendered terms by the authors. In the papers, authors might talk about issues such as gender in relation to leadership or might employ gendered pronouns (e.g. he) when talking about leadership in the abstract (e.g. leadership as male). |
| 1.15 Exclusive | Leadership is described as exclusive by the authors meaning that leadership is not something for everybody. |
| 1.16 Not management | Leadership is explicitly differentiated from management by the authors i.e. authors specifically identify that leadership and management are not necessarily the same but separate entities. |
| 1.17 Followership | Leadership is described as taking a followership role in certain ICU contexts. |
| 1.18 Style | Leadership is defined explicitly as style by the paper authors, including several subthemes.  1.18.1 Leadership defined by authoritarian styles  1.18.2 Leadership defined by democratic styles  1.18.3 Leadership defined by task-orientated styles  1.18.4 Leadership defined by relational styles  1.18.5 Leadership defined by other styles not mentioned above |
| 1.19 Other | Leadership is defined in other ways not captured in codes 1.1-1.18 above. |
| **Leadership discourses** | **Definition** |
| 1.20 Individual discourse | Leadership is seen as something belonging to the individual leader, so the focus is on the leader and his/her behaviours, styles, characteristics, traits etc. |
| 1.21 Contextual discourse | Leadership is seen as something dependent on context with context determining how leadership is enacted such as in the case of situational leadership (where the leader flexes his/her approach to meet the needs of the situation). |
| 1.22 Relational discourse | Leadership is seen as emerging through social interaction between leaders and followers with that relationship based on exchanges (transactional) or leaders inspiring followers to follow (transformational). Leadership is described by the ability to create positive change and relationships in the ICU. |
| 1.23 Leadership as complexity | Leadership is seen as an emergent process occurring within complex adaptive systems, affected by context, systems, relationships and time. Here leadership is distributed across the organisation at all levels. |
| 1.24 Other discourses | Leadership is seen in other ways than the discourses captured in 1.20-1.23 above. |
| **Experiences of leadership:** | **Definition** |
| 2.1 Positive experiences reported by staff | **Here, text relating to positive experiences of leadership in the context of ICU reported by the ICU staff participants will be coded to one or more of the following subthemes:**  **2.1.1 Positive experiences** reported of static leadership processes. This is where the leaders and followers are seen as static often based on traditional workplace hierarchies.  **2.1.2 Positive experiences** reported of emergent leadership processes. This is where leadership emerges depending on context, relationships, time, etc. and may be very different to what might be expected as per static leadership approaches. (Note that this sub-theme may be cross-coded with 1.23 above).  **2.1.3 Positive experiences** reported of leadership as a group or shared process (e.g. shared leadership, distributed leadership etc.). Note that this sub-theme may be cross-coded with 1.22 above.  **2.1.4 Positive experiences** reported of leadership defined by gender (e.g. leadership as male, leadership as female).  **2.1.5 Positive experiences** reported of leadership employing a particular style (e.g. democratic, etc.).  (Note that this sub-theme may be cross-coded with 1.18 above)  **2.1.6 Other** positive experiences reported of leadership not captured by codes 2.1.1-2.1.5 above |
| 2.2 Neutral experiences reported by staff | **Here, text relating to neutral experiences of leadership in the context of ICU reported by the ICU staff participants will be coded to one or more of the following subthemes:**  **2.2.1 Neutral experiences** reported following static leadership process.  **2.2.2 Neutral experiences** reported following emergent leadership process.  **2.2.3 Neutral experiences** reported following leadership as a group or shared process.  **2.2.4 Neutral experiences** reported following leadership defined by gender.  **2.2.5 Neutral experiences** reported following leadership by a particular style.  **2.2.6 Other** neutral experiences reported of leadership not captured by codes 2.2.1-2.2.5 above. |
| 2.3 Negative experiences reported by staff | **Here, text relating to negative experiences of leadership in the context of ICU reported by the ICU staff participants will be coded to one or more of the following subthemes:**  **2.3.1 Negative experiences** reported following static leadership process.  **2.3.2 Negative experiences** reported following emergent leadership process.  **2.3.3 Negative experiences** reported following leadership as a group or shared process.  **2.3.4 Negative experiences** reported following leadership defined by gender.  **2.3.5 Negative experiences** reported following leadership by a particular style.  **2.3.6 Other negative experiences** reported of leadership not captured by codes 2.3.1-2.3.5 above. |
| 2.4 Experiences of leadership against contextual backdrops | **This code relates to the contextual backdrops underpinning ICU staff members’ experiences of leadership expressed in the research papers. These contexts will be coded to the following sub-themes:**  2.4.1 Leadership reported specific to educational roles and processes (e.g. mentoring roles within professions).  2.4.2 Leadership reported specific to assigned management/administrative roles and processes (e.g. leadership as a unit manager).  2.4.3 Leadership reported specific to clinical roles and processes (e.g. intensive care specialist on clinical duty).  2.4.4 Leadership reported specific to non-clinical roles (e.g. director of ICU). |
| **Facilitators to leadership:** | **Definition** |
| 3.1 Individual | **Here, we code text that allude to factors facilitating leadership at the individual level, defined by one or more of the following subthemes:**  3.1.1 Leadership facilitated by leader communication skills.  3.1.2 Leadership facilitated by leader or follower individual styles (e.g. democratic).  3.1.3 Leadership facilitated by previous leader training.  3.1.4 Leadership facilitated by prior leadership and followership experiences.  3.1.5 Leadership facilitated by the age **or seniority** of the leaders and followers.  3.1.6 Leadership facilitated by other factors related to the followers.  3.1.7 Leadership facilitated by other factors related to the individual patient.  3.1.8 Leadership facilitated by other factors related to the leader.  3.1.9. Leadership facilitated by **role allocation.** |
| 3.2 Relational | **Here, we code text that allude to factors facilitating leadership at the relational level, defined by one or more of the following subthemes:**  3.2.1 Leadership facilitated by team structure.  3.2.2 Leadership facilitated by team-based communication.  3.2.3 Leadership facilitated by shared decision-making.  3.2.4 Leadership facilitated by collaboration.  3.2.5 Leadership facilitated by mentoring relationships.  3.2.6 Leadership facilitated by **physical positioning.**  3.2.7 Leadership facilitated by **non-verbal communication.**  **3.2.8** Leadership facilitated by **trust or prior familiarisation**.  3.2.8 Leadership facilitated by another relational factors. |
| 3.3 Organisational | **Here, we code text that allude to factors facilitating leadership at the organisational level, defined by one or more of the following subthemes:**  3.3.1 Leadership facilitated by organisational support (e.g. leader education, time or financial support provided by the organisation).  3.3.2 Leadership facilitated by a positive workplace culture (e.g. staffing allocation, encouragement to speak up).  3.3.3 Leadership facilitated by **protocols or checklists** (e.g. management of a specific crisis).  3.3.4 Leadership facilitated by another organisational factor and not relevant to 3.3.1 to 3.3.3 (e.g. quiet working environment). |
| 3.4 Socio-material | **Here, we code text that allude to factors facilitating leadership at the socio-material level, defined by one or more of the following subthemes:**  3.4.1 Leadership facilitated by **equipment.**  3.4.2 Leadership facilitated by **room layout.**  3.4.3 Leadership facilitated by **clothing.**  3.4.3 Leadership facilitated by **other socio-material factors.** |
| **Barriers to leadership** | **Definition** |
| 3.5 Individual | **Here, we code text that allude to barriers to leadership at an individual level, defined by one or more of the following subthemes:**  3.5.1 Leadership inhibited by pre-existing bias (e.g. cognitive bias).  3.5.2 Leadership inhibited by emotion (e.g. fear of making a poor decision leading to a bad outcome, anger, anxiety, etc).  3.5.3 Leadership inhibited by lack of leader and follower confidence in clinical skills.  3.5.4 Leadership inhibited by lack of leader and follower knowledge.  3.5.5 Leadership inhibited by lack of follower engagement.  3.5.6 Leadership inhibited by poor communication skills of leader.  3.5.7 Leadership inhibited by other individual factors related to the followers.  3.5.8 Leadership inhibited by other factors related to the individual patient.  3.5.9 Leadership inhibited by other factors related to the leader. |
| 3.6 Relational | **Here, we code text that allude to barriers to leadership at a relational level, defined by one or more of the following subthemes:**  3.6.1 Leadership inhibited by unique team structure (e.g. RRT, outer loop communication).  3.6.2 Leadership inhibited by poor team communication.  3.6.3 Leadership inhibited by poor inter-professional collaboration.  3.6.4 Leadership inhibited by **physical positioning.**  3.6.5 Leadership inhibited by lack of **non-verbal communication.**  3.6.6 Leadership inhibited by **noise from other team members.**  3.6.7. Leadership inhibited by other relational factors.  3.6.8. Leadership inhibited by **lack of team trust or familiarisation.** |
| 3.7 Organisational | **Here, we code text that allude to barriers to leadership at an organisational level, defined by one or more of the following subthemes:**  3.7.1 Leadership inhibited by lack of organisational support (e.g. lack of financial support, training or mentoring).  3.7.2 Leadership inhibited by poor workplace culture (e.g. bullying, harassment).  3.7.3 Leadership inhibited by other organisational factors. |
| 3.8 Socio-material | **Here, we code text that allude to barriers to leadership at socio-material level, defined by one or more of the following subthemes:**  3.8.1 Leadership inhibited by **equipment or machines.**  3.8.2 Leadership inhibited by **room layout.**  3.8.3 Leadership inhibited by **clothing.**  3.8.4 Leadership inhibited by **noise from machines/equipment.**  3.8.5 Leadership inhibited by **other socio-material factors.** |
